# A targeted genome association study examining transient receptor potential ion channels, acetylcholine receptors, and adrenergic receptors in Chronic Fatigue Syndrome/Myalgic Encephalomyelitis

**DOI:** 10.1186/s12881-016-0342-y

**Published:** 2016-11-11

**Authors:** Samantha Johnston, Donald Staines, Anne Klein, Sonya Marshall-Gradisnik

**Affiliations:** 1 School of Medical Science, Griffith University, Gold Coast, Australia; 2 The National Centre for Neuroimmunology and Emerging Diseases, Menzies Health Institute Queensland, Griffith University, Southport, Gold Coast, QLD 4222 Australia

**Keywords:** Chronic fatigue syndrome, Myalgic encephalomyelitis, Genome association, Single nucleotide polymorphisms, Adrenergic receptors

## Abstract

**Background:**

Chronic Fatigue Syndrome, also known as Myalgic Encephalomyelitis (CFS/ME) is a debilitating condition of unknown aetiology. It is characterized by a range of physiological effects including neurological, sensory and motor disturbances. This study examined candidate genes for the above clinical manifestations to identify single nucleotide polymorphism (SNP) alleles associated with CFS/ME compared with healthy controls.

**Methods:**

DNA was extracted and whole genome genotyping was performed using the HumanOmniExpress BeadChip array. Gene families for transient receptor potential ion channels, acetylcholine receptors, and adrenergic receptors, and acetylcholinesterase were targeted. The frequency of each SNP and their association between CFS/ME and healthy controls was examined using Fisher’s exact test, and to adjust for multiple testing, False Detection Rate (FDR) and Bonferroni corrections were applied (*p* < 0.05).

**Results:**

The study included 172 participants, consisting of 95 Fukuda defined CFS/ME patients (45.8 ± 8.9; 69 % female) and 77 healthy controls (42.3 ± 10.3; 63 % female). A total of 950 SNPs were included for analysis. 60 significant SNPs were associated with CFS/ME compared with healthy controls. After applying FDR and Bonferroni corrections, SNP *rs2322333* in adrenergic receptor α1 (*ADRA1A*) was higher in CFS/ME compared with healthy controls (45.3 % vs. 23.4 %; *p* = 0.059). The genotype class that was homozygous minor (AA) was substantially lower in CFS/ME compared with healthy controls (4.2 % vs. 24.7 %).

**Conclusions:**

This study reports for the first time the identification of *ADRA1A* and a possible association between CFS/ME and genotype classes. Further examination of the functional role of this class of adrenergic receptors may elucidate the cause of particular clinical manifestations observed in CFS/ME.

## Background

Chronic Fatigue Syndrome, also referred to as Myalgic Encephalomyelitis (CFS/ME) is an illness characterised by chronic, debilitating fatigue that is not alleviated by rest and accompanied by further symptoms. The aetiology of CFS/ME remains unknown however it is associated with a range of physiological impairments including neurological, immunological and autonomic perturbations [1–3]. This includes cognitive difficulties, short-term memory loss, pain, sleep disturbances, sensory and motor disturbances, flu-like symptoms, gastrointestinal disturbances, and autonomic symptoms [3]. Moreover, common infectious events prior to the onset of CFS/ME include respiratory infections and gastrointestinal illness [4].

Biological processes responsible for the varied symptoms reported for CFS/ME may involve several ion channels and receptors that are located on cells throughout the body. Transient receptor potential (TRP) ion channels are widely expressed on tissues and cells and are activated and regulated by various stimuli in the cellular environment such as pain, temperature, taste, pressure, and vision [5]. There are six TRP subfamilies: ankyrin, canonical, melastatin, mucolipin, polycystin, and TRPV [6]. Most consist of non-selective channels permeable to cations such as calcium (Ca^2+^), sodium, and magnesium. This cation permeability has an important role in maintaining homeostasis for a number of physiological requirements. Accordingly, dysregulation of these channels are found to have a role in pathological conditions such as chronic pain, overactive bladder, diabetes, chronic obstructive pulmonary disease, cardiac hypertrophy, familial Alzheimer’s disease, skin diseases, skeletal dysplasias, neuropathy, and cancer [7–12].

In addition to TRP ion channels, acetylcholine receptors (AChRs) are of particular interest due to their role in neurological and neuromuscular transmission [13, 14]. AChRs may have a role in difficulties processing information and short term memory loss reported in CFS/ME [1, 15]. AChRs consist of two types that bind with acetylcholine and transmit its signal. Nicotinic AChRs (nAChRs) are ligand-gated ion channels and are involved in fast synaptic interactions of neurotransmitters [16]. Muscarinic AChRs (mAChRs) consist of 17 different subunits and are G-protein coupled receptors that facilitate slow metabolic responses through secondary messenger cascades [17].

Moreover, adrenergic receptors (ADRs) are another class of G-protein coupled receptors which have catecholamine ligands [18]. This binding is associated with stimulation of the sympathetic nervous system, commonly known for the fight or flight response in which energy is mobilised and blood flow is diverted from non-essential organs to skeletal muscle. There are 3 types of ADRs; Alpha 1 primarily involved in intracellular Ca^2+^ and subsequent smooth muscle contractions [19]. Alpha 2 receptors have a role in inhibition of neurotransmitters, decreased cAMP and decreased smooth muscle contraction. *ß* receptors increase cAMP activity resulting in changes in heart muscle contractions, smooth muscle relaxation and glycogenolysis [20, 21]. All three receptor types have three further subtypes [22].

Previously, we investigated SNPs and genotypes in TRPs and AChRs in peripheral blood mononuclear cells in CFS/ME patients compared with healthy controls [23, 24]. The purpose of this current study was to identify whether further SNPs in TRPs, AChRs, as well as for the first time, ADRs have an association with CFS/ME patients compared with healthy controls. As CFS/ME is largely characterised as a heterogenous illness, this study served to expand our investigation on the above ion channels and receptors due to their wide expression in cells throughout the body and involvement in several physiological processes.

## Methods

### Participants

Participants were from the National Centre for Neuroimmunology and Emerging Diseases (NCNED) research database for CFS/ME. Participants aged between 18 and 65 years were recruited from community support networks in the South East Queensland and Northern New South Wales region of Australia. All participants completed a screening questionnaire reporting their sociodemographic details, medical history, and symptoms. CFS/ME patients were classified according to Fukuda criteria [1]. This required the presence of fatigue that significantly impacts with daily activities for at least 6 months. This should not be due to ongoing exertion or other medical conditions and accompanied by at least four of the following symptoms: post-exertional malaise, unrefreshing sleep, impairment of short-term memory or concentration, muscle pain, joint pain, headaches, tender lymph nodes, and/or sore throat. Healthy controls reported no evidence of disease. Exclusions were participants not meeting the above criteria or with other medical diagnoses that would exclude CFS/ME for example autoimmune disorder, multiple sclerosis, psychosis, major depression, cardiovascular disease. Participants were also excluded if they were pregnant, breast feeding, smokers or had a history of substance abuse.

### DNA extraction

Peripheral blood mononuclear cells were collected into ethylenadiaminetetraacetic acid tubes. Routine pathology was performed for screening of any abnormal parameters including full blood count, erythrocyte sedimentation rate, and high sensitivity C reactive protein by Pathology Queensland. The Qiagen DNA blood mini-kit was used to extract approximately 2 μg of genomic DNA as per manufacturer instructions. To assess the quality and quantity of DNA, the nCounter Digital Analyzer (Nanostring, United States of America) optical scanner was used. Whole genome genotyping was performed using the HumanOmniExpress BeadChip array (Illumina, South Korea).

### Statistical analysis

Statistical analysis was performed using PLINK v1.07 (http://pngu.mgh.harvard.edu/purcell/plink/) whole genome analysis software (Purcell, 2007) to identify the frequency of SNPs. A principle component analysis was performed to identify potential batch effect. For further quality control, a major allele frequency filter of <1 % was applied. Further, SNPs with an effect size variance lower than 2 % were removed. Sample heterozygosity was also applied as a quality control measure and calculated as the proportion of heterozygous genotypes in relation to all genotypes at the SNP and sample levels. Data were compared between CFS/ME patients and healthy controls using R (R Core Team, 2013). Fisher’s exact probability test was used to examine significant genotype association for each individual SNP, and a Bonferroni correction for multiple test correction was applied as post hoc analysis (*p* < 0.05).

### Prediction analysis of significant SNPs

Prediction analysis of significant SNPs were performed using Automated Splice Site and Exon Definition Analysis (http://splice.uwo.ca; ASSEDA). ASSEDA is a web interface, which provides a tool to predict the effects of sequence changes that alter mRNA splicing in human disease. This tool is able to evaluate changes in splice site strength based on theory-based modelling of donor and acceptor splice sites [25].

## Results

### Demographic characteristics

The majority of participants in this study were of Caucasian descent (97.8 %). Of the 172 participants, 95 met criteria for CFS/ME and 77 met criteria for healthy controls, and the mean age and proportion female was 45.8 ± 8.9 (69 % female) and 42.3 ± 10.3 (63 % female) respectively. Potential confounding factors for analysis such as age, sex and ethnicity were analysed for interaction with genes of interest and no outliers were identified, hence no adjustments were required.

### SNP association study

**Fig. 1:**
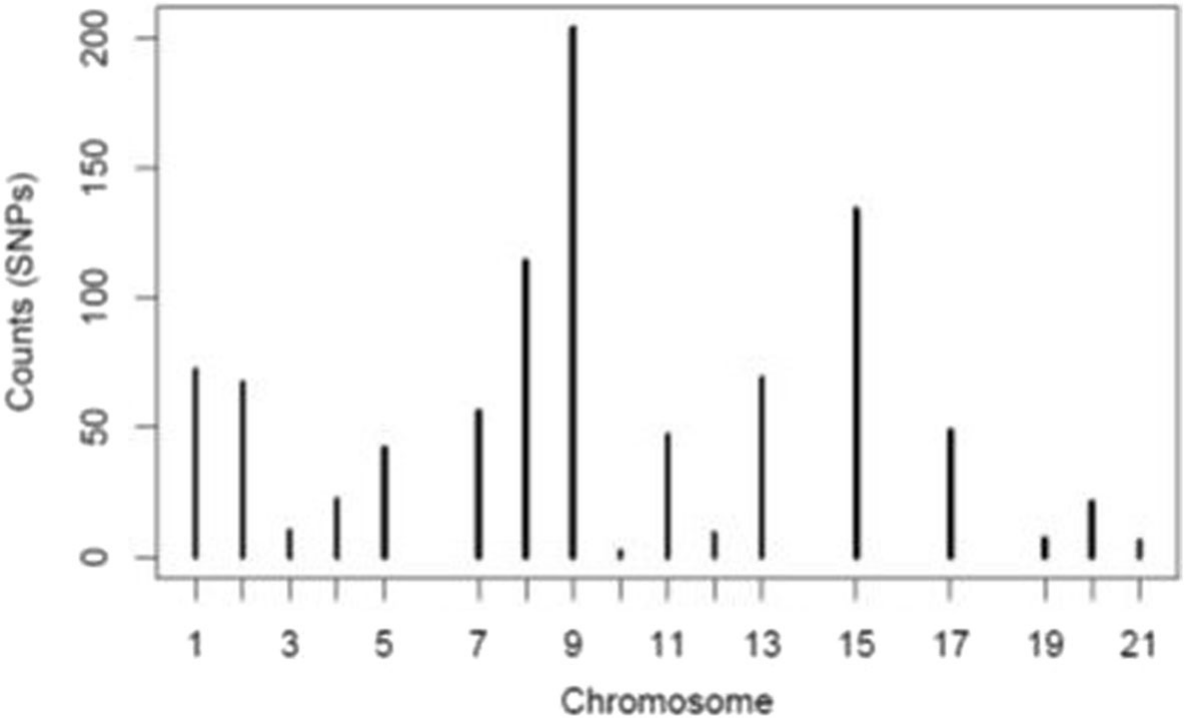
Frequency of SNPs per chromosome

A total of 950 SNPs were included for analysis after quality control measures were applied. The distribution of these SNPs per chromosome is summarised in Fig. 1. Accordingly, the majority of SNPs were observed on chromosome 9 (204 SNPs).

**Fig. 2:**
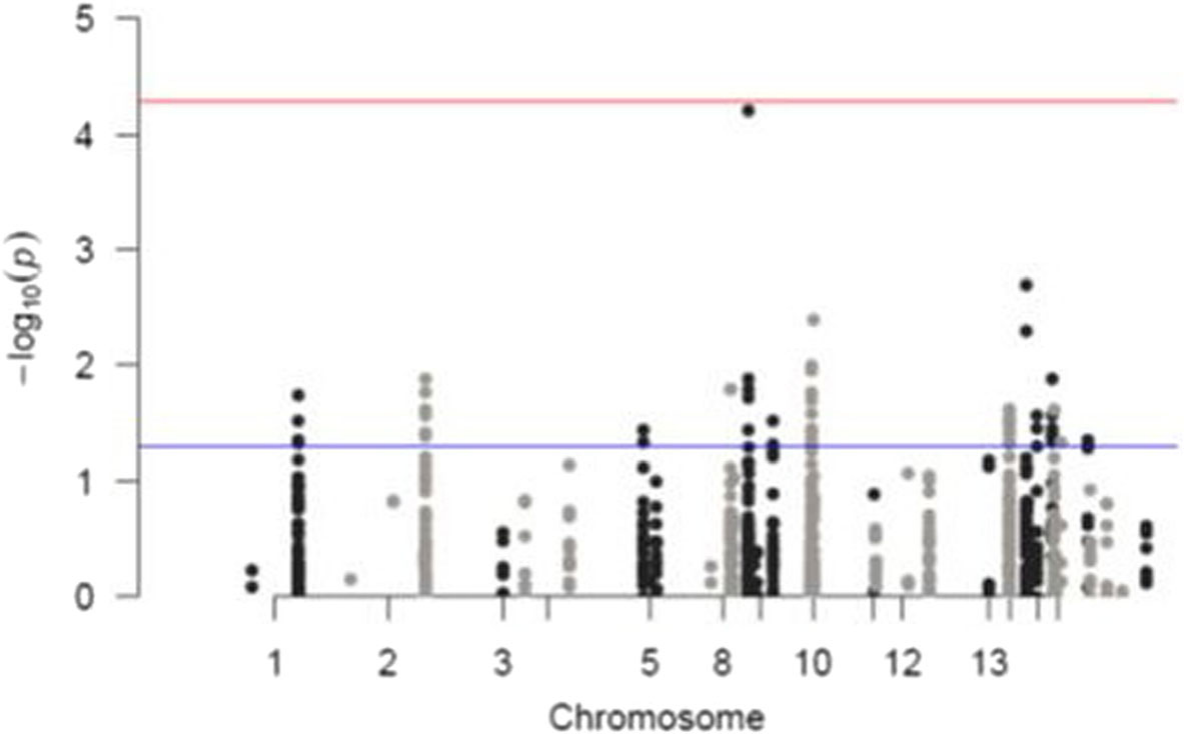
Manhattan plot of Fisher’s exact test on 950 SNPs

Figure 2 demonstrates a Manhattan plot of results of Fisher’s exact test. Blue line corresponds to the significant threshold without any adjustment (raw p-values). Prior to FDR and Bonferroni corrections, 60 significant SNPs were associated with CFS/ME compared with healthy controls. The red line corresponds to the significant threshold after Bonferroni correction.

The raw p-values of the top 10 SNPs identified are summarised in Table 1. The corresponding frequencies in CFS/ME compared with healthy controls are shown. Following adjustment applying FDR and  Bonferroni corrections, the association with alpha 1A ADR (*ADRA1A*) SNP *rs2322333* located on chromosome 8 was almost significant (*p* = 0.059). The proportion of CFS/ME patients homozygous major (GG) for this SNP was higher compared with healthy controls. Moreover, the genotype class that was homozygous minor (AA) was much lower in CFS/ME patients compared with healthy controls (4.2 % vs. 24.7 %).

**Table 1:** Results of Fisher’s exact test for top 10 SNPs

Gene	SNP name	raw *p*-value	padj FDR	padj Bonferroni	Genotype	Controls allele frequency (%)	Cases allele frequency (%)	Odds ratios
ADRA1A	rs2322333	6.2e-05	0.059	0.059	AA	19	4	0.08
					AG	40	48	0.5
					GG	18	43	1
TRPM1	rs4779824	0.002	0.788	1	CC	34	4	0.02
					TC	39	51	0.26
					TT	4	20	1
TRPM6	rs11787707	0.004	0.788	1	AA	54	84	1
					AG	22	11	0.32
					GG	1	0	0
TRPM1	rs10467996	0.005	0.788	1	CC	4	17	5.72
					TC	34	49	1.94
					TT	39	29	1
TRPM3	rs10118380	0.01	0.788	1	CC	8	21	3.39
					TC	52	43	1.07
					TT	31	24	1
TRPM3	rs7022747	0.013	0.788	1	AG	8	1	NA
					GG	69	94	10.9
					AA	NA	NA	NA
CHRNB4	rs1316971	0.013	0.788	1	AA	10	0	0
					AG	38	32	0.65
					GG	46	60	0
ADRA1A	rs526302	0.013	0.788	1	GG	39	63	1
					TG	29	30	0.64
					TT	9	2	0.14
TRPM8	rs6719311	0.013	0.788	1	AA	0	0	NA
					AG	20	8	0.37
					GG	68	74	NA
ADRA1A	rs11782159	0.016	0.788	1	AA	29	21	1
					AC	39	50	1.77
					CC	9	24	3.68

*Abbreviations*: *SNP* single nucleotide polymorphism, *padj* adjusted *p*-value, *FDR* false detection rate, *ADRA* adrenergic receptor alpha, *TRPM* transient receptor potential melastatin ion channel, *CHRN* cholinergic receptor nicotinic beta

### Prediction analysis

ASSEDA analysis predicted that a mutation of rs2322333, located in the intronic and 3′UTR part of the gene ADRA1A in chromosome 8, has a probability of being deleterious with a ΔRi = −9.4, fold change = −32.7 and ΔZ = −2.8. This mutation leads to the abolition of ELAV-like protein 1 (ELAVL1) binding site rs2695260.

## Discussion

This study is the first to identify *ADRA1A* as a novel candidate gene for CFS/ME. After stringent corrections for multiple testing were applied, the *ADRA1A* SNP remained predominant. Moreover, the proportion of patients that were homozygous minor, AA was much lower in CFS/ME compared with healthy controls. These results particularly suggest that patients exhibiting this allele marker may have a decreased risk of development of CFS/ME.

The specific physiological implications of *ADRA1A* are mainly involved in smooth muscle contraction [20]. This is required for vasoconstriction of blood vessels throughout the body including the skin, gastrointestinal system, genitourinary system, kidney and brain. It is also involved in the glycogenolysis and gluconeogenesis of adipose tissue in the liver, in addition to enabling secretions from sweat glands [26–29]. These above processes have been associated with symptomatology of CFS/ME [3, 4]. Hence, the differential expression of *ADRA1A* may explain particular clinical phenotypes of CFS/ME.

Alpha 1 ADRs are members of the superfamily for G protein-coupled receptors [30]. When activated, heterotrimeric G protein (G_q/11_) in turn activates phospholipase (PLC). PLC cleaves phosphatidylinositol 4,5-biphosphate, which leads to an increase in inositol triphosphate (IP_3_) and diacylglycerol (DAG). IP_3_ acts as a secondary messenger and is a soluble molecule that is able to diffuse through the cytoplasm to the endoplasmic reticulum of cells (or sarcoplasmic reticulum in muscle cells) to stimulate Ca^2+^ influx. This process involves the binding of IP_3_ ligand to IP_3_ sensitive Ca^2+^ channels that result in the release of Ca^2+^ into the cytoplasm [31, 32]. This mechanism contributes to a number of cellular processes, including a slow after depolarizing current (sADP) in neurons [33].

Of particular interest is the functional role of the SNP *rs2322333* identified in this study. *ADRA1A* is coded from eleven variants where the SNP *rs2322333* is located in the 3′ untranslated region (3′-UTR) of two of these variants, namely, *ENST00000276393*, *ADRA1A-003* and *ENST00000380572 ADRA1A-005*, while the remainder are located in the intronic region of this gene (http://ensembl.org). The 3′-UTR is a binding site for regulatory proteins, binding to specific sites within the 3′-UTR may decrease gene expression of various mRNAs by either inhibiting translation or directly causing degradation of the transcript. [34] Moreover, the allele AA for *rs2322333* may have a key role in ligand selectivity [22].

Through prediction analysis, this study identified that mutation of rs2322333 in *ADRA1A*, results in the abolition of an RNA binding protein, *ELAVL1*. *ELAVL1* is known to bind to the 3′-UTR region of mRNAs to increase stability [10–15]. A lack of these proteins can alter the splicing of DNA, consequently changing protein expression by a change in isoforms. In particular, *ELAVL1* has been found to bind with adenolate-uridylate rich elements (AREs) in c-fos mRNAs [12]. The c-fos mRNAs are involved in several cellular processes that include cell proliferation, survival and differentiation. It further has a role in the physiological processes of oxygen deficiency in the tissues (hypoxia) and the formation of new blood vessels (angiogenesis) [16]. Hence, dysregulation of c-fos mRNAs are known to have an important factor in cancer development.

Furthermore, *ELAVL1* bind with AREs in interleukin-3 (IL-3) mRNAs. IL-3 is an important cytokine that promotes cell growth. It stimulates the differentiation of hematopoietic stem cells into myeloid progenitor cells or with IL-7, into lymphoid progenitor cells. In combination with other cytokines IL-3 stimulates the proliferation of all myeloid cells including granulocytes, monocytes, and dendritic cells. In autoimmunity, IL-3 and other cytokines are important for the proliferation of regulatory T cells (Tregs) [17]. Dysfunction of Tregs has been reported in immunological dysfunction of CFS/ME, in which FoxP3 secretion by CD4+ T cells has been found to be significantly higher in patients compared with healthy controls [18, 19].

Moreover, *ELAVL* *1* with zinc finger protein 385A (ZNF385A), another RNA-binding protein, can affect the localization and translation of p53/TP53 mRNA. With ZNF385A, *ELAVL1* binds the 3′-UTR of p53/TP53 mRNA to control their nuclear export induced by cyclin dependent kinase inhibitor 2A (CDKN2A), which is a protein coding gene. This may regulate p53/TP53 expression and have a role in the mediation of CDKN2A anti-proliferative activity. Accordingly, the dysregulation of CDKN2A is associated with the development of a wide variety of tumours. Alternatively, it may also regulate p53/TP53 activity through direct protein-protein interaction. This may promote cell-cycle arrest over apoptosis, through preferential binding of DNA and transactivation of p53/TP53 on cell-cycle arrest target genes over pro-apoptotic target genes. It may also regulate the ubiquitination and stability of cyclin dependent kinase inhibitor 1A (CDKN1A), which inhibits cellular proliferation in response to DNA damage [20].

Physiologically, ADR subtypes are associated with numerous systems including neurological, cardiovascular, respiratory and urogenital systems. [27, 35] Alpha 1 ADRs exert effects in hypothalamo-pituitary-adrenocortical (HPA) axis, corticotropin releasing factor (CRF) and behavioural stress responses [36]. Alpha 1 ADRs are widely distributed in CNS including neurons, glial cells and located in hypothalamic nuclei, brainstem and spinal cord notably associated with motor function [36].

Alpha 1 ADRs are located in the nuclei of adult myocardium cells and participates in intra-nuclear signalling [37]. These receptors regulate cardioprotective functions including physiological hypertrophy, protection from apoptosis, positive iontropy and preconditioning. Importantly alpha 1 ADRs rescue myocytes from cell death via an alpha 1A-ERK signalling pathway. Rescue is mimicked by expression of (mitogen activated protein kinase kinase) MEK which increases (extracellular signal-related kinase) ERK activity [38]. We have recently reported significant changes in ERK1/2 and MEK 1/2 in isolated natural killer cells from CFS/ME patients. Specifically, we reported a significant reduction in ERK1/2 and also a significant increase in MEK1/2, suggesting a dysregulation in MEK/ERK signalling not only in NK cells but also conceivably in cardiac myocytes and other tissues that have these receptors [39].

O’Connell et al. [40], also describe alpha 1 ADRs in nuclei of human heart myocardium and their role in cardiac failure. These authors assert that these receptors have a strongly cardioprotective role in prevention of cardiac myocyte death through activation of ERK. Alpha 1 ADRs can participate in myocardial contractile function both through intrinsic inotropic effects as well as countering the negative inotropic response mediated by other subtypes [41]. It is likely that alpha 1 ADRs also participates in protection from hypoxia. Compromise of function could potentially contribute to adverse symptomatology associated with impaired neuroprotection resulting from chronic intermittent hypobaric hypoxia (CIHH). CIHH increases the activity of alpha 1 ADRs, which is possibly one of the mechanisms for the cardioprotection of CIHH [42]. Importantly, the cardiovascular system in CFS/ME has been reported to be compromised as manifested by postural orthostatic tachycardia syndrome, poor peripheral circulation, intolerance to physical exercise and poor cerebral perfusion and systemic perfusion [43–47]. Collectively, these symptoms and signs suggest a clinical presentation of dysautonomia.

This study further expands our previous investigations into TRPs, AChRs, and ADRs in CFS/ME. In particular, it is the first genome association study on adrenergic receptors conducted on an Australian cohort with CFS/ME. A particular strength of this study was the considerable association with *ADRA1A* gene found among a preliminary cohort of patients, when strict statistical considerations were applied. Further validation of this and previous preliminary findings in TRPs and AChRs, will be required in a replication study in a larger cohort of patients. However, the present findings provide supporting evidence for investigation into the role of adrenergic receptors in CFS/ME.

## Conclusions

In conclusion, this study demonstrated that the *ADRA1A* gene may be a potential cellular marker for CFS/ME. It is recommended that future studies examine the role of adrenergic genes and specifically their genotypes to elucidate whether they have a potential protective role against CFS/ME.

## Abbreviations

3′-UTR: 3′ untranslated region
AChR: Acetylcholine receptor
ADR: Adrenergic receptor
ADRA1A: Adrenergic receptor alpha 1A
Ca^2+^: calcium
cAMP: Cyclic adenosine monophosphate
CFS/ME: Chronic fatigue syndrome/myalgic encephalomyelitis
CIHH: Chronic intermittent hypobaric hypoxia
DAG: Diacylglycerol
DNA: Deoxyribonucleic acid
ERK: Extracellular signal regulated kinase
IP_3_: Inositol triphosphate
MAChR: Muscarinic acetylecholine receptor
MEK: Mitogen activated protein kinase kinase
NAChR: Nicotinic acetylcholine receptor
NCNED: National centre for neuroimmunology and emerging diseases
NK: Natural killer
PLC: Phospholipase C
SNP: Single nucleotide polymorphism
TRP: Transient receptor potential
